# The Complement Cascade as a Mediator of Human Malignant Hematopoietic Cell Trafficking

**DOI:** 10.3389/fimmu.2019.01292

**Published:** 2019-06-07

**Authors:** Anna Lenkiewicz, Kamila Bujko, Katarzyna Brzezniakiewicz-Janus, Bing Xu, Mariusz Z. Ratajczak

**Affiliations:** ^1^Department of Regenerative Medicine, Center for Preclinical Research and Technology, Warsaw Medical University, Warsaw, Poland; ^2^Stem Cell Institute at James Graham Brown Cancer Center, University of Louisville, Louisville, KY, United States; ^3^Department of Hematology, Multi-Specialist Hospital Gorzow Wlkp., University of Zielona Gora, Zielona Gora, Poland; ^4^Department of Hematology, The First Affiliated Hospital of Xiamen University and Institute of Hematology of Xiamen University, Xiamen, China

**Keywords:** complement cascade, leukemia, chemotaxis, cell migration, inflammasome, Hmgb1, S100a9, HO-1

## Abstract

The complement cascade (ComC) cleavage fragments C3a and C5a regulate the trafficking of normal, differentiated hematopoietic cells, although they do not chemoattract more primitive hematopoietic stem/progenitor cells (HSPCs). By contrast, human myeloid and lymphoid leukemia cell lines and clonogenic blasts from chronic myelogenous leukemia (CML) and acute myelogenous leukemia (AML) patients respond to C3 and C5 cleavage fragments by chemotaxis and increased adhesion. Consistent with this finding, C3a and C5a receptors are expressed by leukemic cells at the mRNA (RT-PCR) and protein (FACS) levels, and these cells respond to C3a and C5a stimulation by phosphorylation of p44/42 MAPK and AKT. However, neither of these ComC cleavage fragments have an effect on cell proliferation or survival. In parallel, we found that inducible heme oxygenase 1 (HO-1)–an anti-inflammatory enzyme, is a negative regulator of ComC-mediated trafficking of malignant cells and that stimulation of these cells by C3 or C5 cleavage fragments downregulates HO-1 expression in a p38 MAPK-dependent manner, rendering cells exposed to C3a or C5a more mobile. We propose that, while the ComC is not directly involved in the proliferation of malignant hematopoietic cells, its activation in leukemia/lymphoma patients (e.g., as a result of accompanying infections or sterile inflammation after radio-chemotherapy) enhances the motility of malignant cells and contributes to their dissemination in a p38 MAPK–HO-1 axis-dependent manner. Based on this idea, we propose that inhibition of p38 MAPK or upregulation of HO-1 by available small-molecule modulators would have a beneficial effect on ameliorating expansion and dissemination of leukemia/lymphoma cells in clinical situations in which the ComC becomes activated. Finally, since we detected expression of C3 and C5 mRNA in human leukemic cell lines, further study of the potential role of the complosome in regulating the behavior of these cells is needed.

## Introduction

Leukemia that is resistant to standard chemotherapy or that recurs after myeloablative treatment and transplantation of hematopoietic stem/progenitor cells (HSPCs) remains an important clinical problem. This malignancy is first initiated locally in hematopoietic tissue and, depending on the type of leukemic cells, has the tendency to spread to other areas of bone marrow (BM), spleen, or lymph nodes and may finally infiltrate solid organs, such as liver, brain, or skin. The origin of cells that initiate leukemia is still disputed and may include mutated HPSCs or even more differentiated cells from hematopoietic or lymphoid lineages (1–3).

Since leukemic cells express several receptors that are also functional on the surface of normal hematopoietic cells and that regulate migration and adhesion, they are highly responsive to similar growth factors, cytokines, chemokines, bioactive phospholipids, and extracellular nucleotides (EXNs) that direct migration of normal hematopoietic cells (3, 4). These pro-migratory factors are already expressed in the hematopoietic microenvironment under steady-state conditions and, what is important here, can be upregulated in response to tissue-damaging chemotherapy or radiotherapy. Upregulation of these pro-migratory factors leads to unwanted side effects—namely, metastasis to hematopoietic and non-hematopoietic organs (5, 6).

We will present here the concept that chemo/radiotherapy-induced tissue/organ damage is a form of sterile inflammation that involves activation of innate immunity. According to the definition, sterile inflammation is an inflammatory process triggered in the absence of microbial pathogens; however, most of the innate immune pathways that sense microbial infections are also involved in this process (7, 8). For example, chemotherapy- or radiotherapy-induced cell damage leads to release of several danger-associated molecular pattern molecules (DAMPs) that may induce the innate immune response by activating the complement cascade (ComC) (7, 9). DAMPs released from damaged cells activate the ComC in a mannan-binding lectin (MBL)–mannan associated protease (MASP)-dependent manner. As a consequence, MASP-1/2 activates downstream elements of the ComC but in parallel also triggers activation of the coagulation cascade (CoaC) and the fibrinolytic cascade (FibC) (10–12). DAMPs, depending on their molecular structure, may also bind to the family of Toll-like receptors (TLRs). One of the most important DAMPs released from damaged cells is extracellular ATP, which binds to P2 purinergic receptors and, after binding to the P2X7 receptor, activates the inflammasome (13).

Based on this mechanism, a chemotherapy or radiotherapy-induced pro-inflammatory microenvironment in hematopoietic organs and other tissues leads to release of several peptide- and non-peptide-based mediators, including bioactive lipids and ExNs, such as ATP, and activate the three ancient cross-interacting proteolytic cascades, the ComC, CoaC, and FibC (5, 6, 9, 13, 14).

In this review we will focus on the key role of the ComC and address the most important pathways that lead to its activation after chemo/radiotherapy. First, we will focus on the important role of the MBL pathway of ComC activation, which is triggered by release of DAMPs and reactive oxygen species (ROS) from damaged cells (11–13). Next, we will highlight the role of EXNs, and in particular ATP, in activating the inflammasome and the release from cells of IL-1β, IL-18, and several DAMPs, including high mobility group box 1 (Hmgb1) and calgranulin B (S100a9) (15). In particular, Hmgb1, as an important DAMP, is recognized by the circulating pattern-recognition receptor MBL, and the Hmgb1–MBL interaction potentiates activation of the ComC in an MBL–MASP-dependent manner (6).

Moreover, since the ComC and sterile inflammation can be inhibited by anti-inflammatory treatment, including upregulation of heme oxygenase 1 (HO-1) in the cells or downregulation of the inflammasome, we will also address the potential application of HO-1-activating molecules or inflammasome inhibitors in ameliorating a chemotherapy-induced pro-metastatic microenvironment (16, 17). Chemotherapy/radiotherapy-induced sterile inflammation in collateral tissues could also be easily ameliorated after administration of non-steroid inflammatory drugs or anti-inflammatory steroids (5, 6).

### Response of Normal HSPCs to ComC Activation and Stimulation by C3 and C5 Cleavage Fragments

Receptors for soluble C3 and C5 cleavage fragments (C3aR and C5aR, also known as C5aR1 or CD88) are expressed by normal HSPCs as well as several differentiated cells from hematopoietic and lymphatic lineages, including leucocytes, monocytes, lymphocytes, and dendritic cells (7, 9, 12). Both C3a and C5a are reported to be potent activators and chemo attractants for mast cells, granulocytes, and monocytes and, as anaphylatoxins, play an important role in activation and degranulation of granulocytes. Both C3a and C5a are degraded to _desArg_C3a and _desArg_C5a, and the second of these retains significant biological activity. However, neither C3a nor C5a chemoattract normal HSPCs, C3a may enhance the responsiveness of these cells to stromal-derived factor 1 (SDF-1), an important α-chemokine playing a role in retention of HSPCs in BM niches as well as directing the homing of HSPCs after transplantation to the BM. This effect relies on a C3a-mediated increase in incorporation of the SDF-1 receptor, CXCR4, into membrane lipid rafts (18). Evidence has accumulated that CXCR4, if incorporated into membrane lipid rafts, responds much more strongly to an SDF-1 gradient, which is explained by the fact that lipid rafts are membrane domains associated with downstream signaling molecules involved in signal transduction from activated CXCR4. This has been demonstrated by direct confocal colocalization studies at the single-HSPC level and by western blot analysis demonstrating the presence of CXCR4 in lipid raft-enriched cell membrane fractions. As a result, HSPCs briefly exposed (primed) with C3a before transplantation into lethally irradiated animals show enhanced seeding efficiency to BM niches (19). This proposed strategy could potentially be employed in the clinic to facilitate engraftment of transplanted HSPCs and to accelerate hematopoietic recovery after the procedure. Thus, while ComC cleavage fragments do not directly chemoattract normal HSPCs, they play an important role in enhancing the responsiveness of these cells to a retention/homing SDF-1 gradient.

Moreover, ComC cleavage fragments play a pivotal role in mobilization of HSPCs from BM into peripheral blood (PB), as seen for example in inflammation, tissue/organ injury, or during pharmacological mobilization—a clinical procedure to harvest for transplantation purposes normal HSPCs that are mobilized from BM into PB (18, 19). In this procedure, pro-mobilizing agents, such as cytokine granulocyte colony stimulating factor (G-CSF), activate granulocytes to release several proteolytic and lipolytic enzymes, ROS, and DAMPs, which facilitate release of HSPCs from their BM niches. This effect is subsequently enhanced in a positive feedback loop by terminal products of ComC activation, C5a, and to some extent _desArg_C5a. Granulocytes activated by C5a are subsequently chemo attracted to a C5a gradient present in PB and are the first cells that egress from BM into PB during mobilization and thus pave the way for HSPCs to follow in their footsteps across the PB–BM endothelial barrier (18–21).

**Figure 1:**
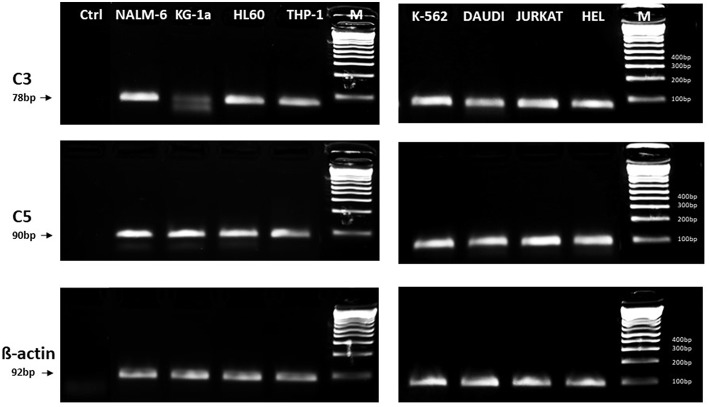
Expression of C3 and C5 mRNA in various myeloid and lymphoid leukemia cell lines. Expression was detected in purified mRNA samples by reverse transcription polymerase chain reaction (RT-PCR). Samples containing only water instead of cDNA were used in each run as negative controls. Representative agarose gels of the RT-PCR amplicons are shown. Sequence of primers employed is shown in Supplementary Materials.

In light of the most recent observations, the role of the ComC in regulating cell biology, including that of hematopoietic stem cells, may seem even more surprising. Namely, in addition to the long-prevailing classical view of the ComC as a serum-operative danger sensor and first line of defense system in the organism, a novel concept has been recently proposed in which the ComC regulates the biology of normal stem cells in an autocrine-dependent manner. In support of this notion, experimental evidence has accumulated that C3 is present inside cells as a component of the “complosome,” and its activation may impact cell biology (22–24). Thus, further studies are needed to better understand the role of the complosome in normal and malignant hematopoiesis. Intracellularly expressed autocrine C3a may mask the responsiveness of exogenously added C3a as a stimulating molecule in several biological assays. Confirming that this is a potential complication, we detected the presence of C3 and C5 by PCR in normal human and murine hematopietic stem cells and in several leukemia cell lines (Figure 1). Therefore, further work is needed to understand the implications of the endogenous expression of C3 both in normal and in malignant hematopoietic cells (25).

In summary, despite the fact that normal human HSPCs express C3a and C5a receptors and, after being primed by C3a, respond significantly more strongly to an SDF-1 gradient, surprisingly, they do not show spontaneous chemotaxis in response to C3 and C5 complement cleavage fragments. This is in contrast to immortalized leukemia and lymphoma cell lines and clonogenic leukemic progenitors, which will be discussed below in this review.

### Induction of a Pro-metastatic Microenvironment in BM and Other Organs in Response to Chemotherapy in a Sterile Inflammation-Dependent Manner

Accumulating evidence shows that leukemia contains some rare, primitive (cancer stem cell-like) cells that are highly mobile and, if they survive applied chemotherapy or a myeloablative procedure prior to hematopoietic transplantation, are responsible for leukemia regrowth (1–3). These recurring leukemic cells, like normal hematopoietic cells, respond by chemotaxis to several chemo attractants present in hematopoietic organs, PB, and lymph as well as to those upregulated in pre-metastatic niches in solid organs (3, 4, 13, 14). Therefore, this chemotactic microenvironment, creating a fertile soil for metastasizing leukemic cells, is induced as an unintentional side effect of chemotherapy. A well-known example of such a phenomenon is that SDF-1, an important chemottractant for normal and malignant hematopoietic cells, as mentioned above, is upregulated in BM after conditioning for transplantation by myeloablative administration of cytostatics or irradiation (26). It is known that upregulation of SDF-1 in myeloablated BM along with other factors is required for homing and engraftment of HSPCs infused into PB during hematopoietic transplantation. Simiarly, SDF-1 induced in BM after radio/chemotherapy applied for other non-transplantation-related reasons, such as systemic chemotherapy, has been postulated to promote metastasis of therapy-resistant cancer cells to the bones (27).

In addition to SDF-1, chemotherapy induces expression of several other chemo attractants in the BM microenvironment, including bioactive phospholipids, such as sphingosine-1-phosphate (S1P) and ceramide-1-phosphate (C1P), and releases EXNs, including ATP, from damaged cells, which may all chemoattract solid tumor and leukemia cells (3–6). This unwanted effect seen in BM is a result of therapy-induced sterile inflammation in tissues exposed to cytostatics or irradiation and could be ameliorated by anti-inflammatory treatment. Our group has already demonstrated this phenomenon in more detail in the metastasis of solid tumors, with evidence that simple anti-inflammatory treatment of experimental animals with relatively simple compounds, such as non-steroid anti-inflammatory drugs (e.g., ibuprofen) or steroids (e.g., dexamethasone), given shortly after chemotherapy ameliorates induction of pro-metastatic niches in various organs and reduces the metastatic spread of intravenously infused cancer cells (5, 6).

In summary, by inducing a prometastatic microenvironment, chemotherapy or irradiation could be a double-edged sword that limits the therapeutic benefits of anti-cancer treatment. More importantly, this concept applies also to leukemia, and we will address this issue below.

### Activation of the ComC in Response to Chemotherapy Due to Induction of Sterile Inflammation Mediated by Activation of the Inflammasome

It is known that the ComC plays a role in the pathogenesis of several solid tumors by modifying their growth, adhesion, affecting metastatic potential, and affecting their response to therapeutics (28). By contrast, much less information has been reported on the role of the ComC in the pathogenesis and progression of leukemia, and lymphoma and cancer cells may become exposed to ComC cleavage fragments, as seen, for example, during sterile or microbe-mediated inflammation (17).

We propose that activation of the ComC after chemotherapy or irradiation occurs in patients via DAMP-induced sterile inflammation (3, 8, 29). We also propose that the inflammasome, and in particular one of its family members, NLRP3, is actively involved in this process. Overall, inflammasomes as mentioned above are intercellular multimeric complexes, and the NRLP3 inflammasome seems to play the most important role in the response of this family to a variety of physiological and pathogenic stimuli. Activation of the NRLP3 inflammasome complex leads to cellular release of IL-1β and IL-18, which are activated by proteolytic processing to active forms by caspase 1 before secretion. In a parallel result of inflammasome activation, cells release any of several DAMPs, including the abovementioned Hmgb1, which are recognized by MBL and directly activate the ComC in an MBL-dependent manner (15). However, one has to remember that the functional output of the inflammasome is much broader and, after sufficiently strong activation, may induce an inflammatory form of cell death called pyroptosis. Thus, as recently proposed, the inflammasome operates inside cells at the intersection of the inflammatory response with fundamental cellular processes, including cell death (15).

**Figure 2:**
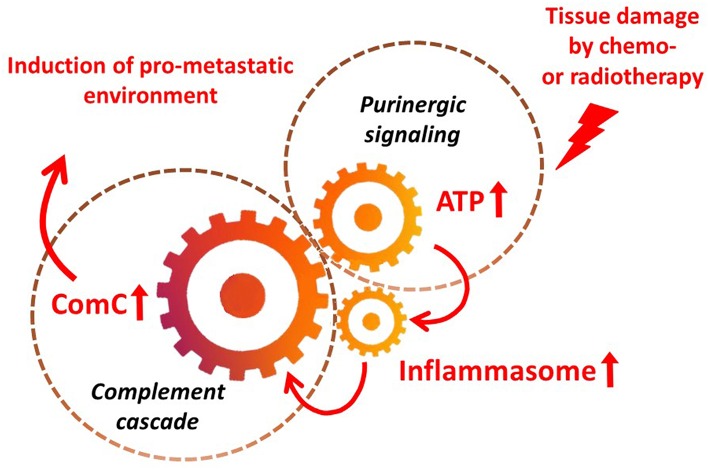
The inflammasome as a “cogwheel” coupling purinergic signaling with the complement cascade. As proposed, tissue damage after exposure to chemotherapy or irradiation activates release of several mediators from damaged tissues, including ATP. As part of purinergic signaling, ATP activates P1 receptors, including the P2X7 receptor, on the surface of bone marrow mononuclear cells and stromal cells, which activates the inflammasome. Danger-associated molecular pattern molecules (DAMPs; e.g., Hmgb1) released from cells in which the inflammasome has become activated and from other cells directly damaged by chemo- or radiotherapy activate the complement cascade (ComC) in an MBL–MASP-dependent manner. Activation of the ComC maintains sterile inflammation in damaged tissues.

It is widely accepted that a crucial mediator activating the NLRP3 inflammasome in hematopoietic cells is extracellular ATP, which is one of the crucial components released, as mentioned above, in response to stress related to tissue or organ injury, as seen for example after chemotherapy or irradiation (3, 11, 13). As an extracellular signaling molecule, ATP activates several purinergic G protein-coupled receptors on the surface of cells, and P2X7 receptor activation is particularly crucial in triggering activation of the NRLP3 inflammasome response. In this review, we present the concept that the NRLP3 inflammasome acts as a “cogwheel” that couples a purinergic signaling mediator, ATP, which is released from damaged cells, with activation of the MBL pathway of the ComC (Figure 2).

**Figure 3:**
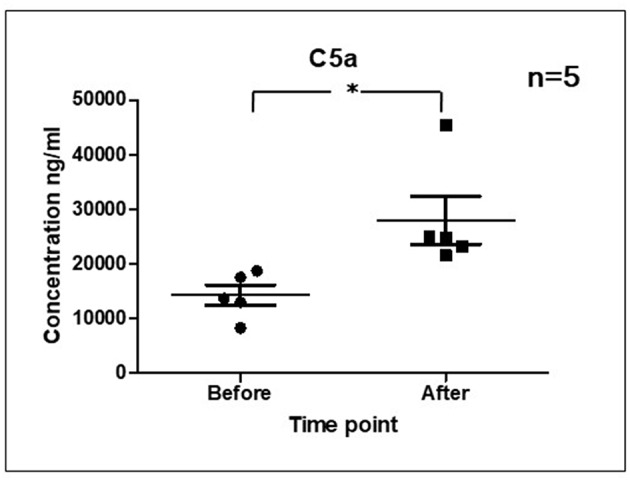
Activation of ComC in patients undergoing chemotherapy. Patient serum samples were isolated from 5 primary AML patients peripheral blood (4°C/4,000 g, 15 min) before or 24 h after chemotherapy, respectively. All patients received “3+7” IA (IDA+Ara-C) regimens induction treatment. Serum complement 5a(C5a) level was measured by ELISA assay (Human complement C5a ELISA Kit; SAB Cat No. EK5444). Data represent the mean value ± SEM for two independent experiments. ^*^p < 0.05; (independent-sample *t*-test).

**Figure 4:**
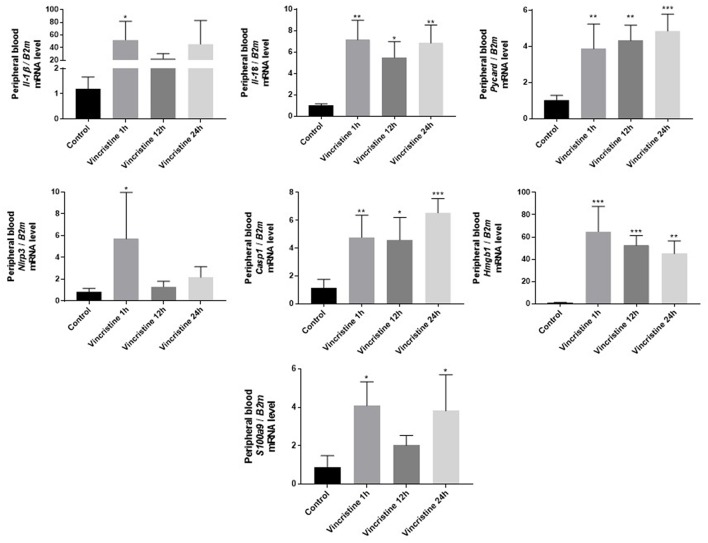
Expression of genes involved in inflammasome activation and propagation in mouse peripheral blood after 1-, 12-, or 24-h vincristine treatment. Expression of genes encoding interleukin-1β (*Il-1β*), interleukin-18 (*Il-18*), Asc (*Pycard*), NLRP3 (*Nlrp3*), caspase 1 (*Casp1*), high mobility group box 1 (*Hmgb1*), and calgranulin B (*S100a9*) in mouse peripheral blood after 1-, 12-, or 24-h treatment with 0.5 mg/kg vincristine, as measured by qRT-PCR. Results were normalized to the β2 microglobulin (*B2m*) level. Data represent the mean value ± SEM for four independent experiments. ^*^p < 0.05; ^**^p < 0.001; ^***^p < 0.001 compared with control (one-way ANOVA followed by Bonferroni *post hoc* test). Sequence of primers employed is shown in Supplementary Materials.

As it is shown in Figure 3 we confirmed activation of ComC in that AML patients exposed to chemotherapy. Next, to demonstrate involvement of the inflammasome in the ComC response to chemotherapy-induced sterile inflammation, we exposed mice to vincristine. We then evaluated (i) ELISA activation of the ComC by detecting the C5a cleavage fragment in PB and (ii) activation of inflammasome components in PB-derived mononuclear cells by looking for upregulation of the mRNA level for genes encoding interleukin-1β (*Il-1*β), interleukin-18 (*Il-18*), Asc (*Pycard*), NLRP3 (*Nlrp3*), caspase 1 (*Casp1*), high mobility group box 1 (*Hmgb1*), and calgranulin B (*S100a9*) (Figure 4). We confirmed that, like the response to radiotherapy (30), the inflammasome is activated by exposing experimental animals to cytostatic treatment. Our results also suggest that DAMPs released from the cells (e.g., Hmgb1), as known ligands for MBL, activate the ComC in an MBL-dependent manner (7, 11, 12). Activation of the ComC and release of C3a and C5a initiates several processes involving activation of innate immunity cells and release of proteolytic and lipolytic enzymes. Moreover, the C3a and C5a ComC cleavage products maintain a positive amplification loop to sustain the sterile inflammation response.

Thus, as proposed in Figure 2, and supported by our results, activation of the inflammasome in an ATP-dependent manner and the release of DAMPs seems to be an important mechanism of ComC activation in response to chemotherapy. The same mechanism seems to operate after irradiation (30). Nevertheless, the inflammasome, in addition to ATP, may also be activated by other factors released in response to chemotherapy or irradiation, such as S1P (3, 5, 6). On the other hand, the ComC could also be activated by other mechanisms in leukemic patients who suffer from accompanying infections as a response to pathogen-associated molecular pattern molecules (PAMPs), which also trigger the classical and alternative pathways of ComC activation.

In addition, as with normal hematopoietic cells, further studies are needed to shed more light on the potential role of inflammasome activation in directly regulating biological processes in human leukemic blasts (31). It is also important to investigate the interplay of inflammasome activation with the intracellular C3 and C5 complesome (22–24). In fact, intracellular C5 activation has been shown to be required for NLRP3 inflammasome assembly in human CD4^+^ T lymphocytes, and this is modulated by the differential activation of C5aR vs. the surface-expressed alternate receptor C2L2 (C5aR2) (32). In further support of such a mechanism, we found, as mentioned above, that human leukemia cells lines express endogenous mRNA for C3 and C5 (Figure 1) and express several elements of the inflammasome complex (not shown). It is worth mentioning that there have been initial attempts to modulate activity of the inflammasome in leukemic cells by employing small-molecule inhibitors of this pathway (33). Such treatments may have a positive effect on inhibiting leukemia cell progression and spread, and it has been reported that NLRP3 overexpression or activation inhibits cell proliferation and stimulates apoptosis in chronic lymphocytic leukemia cells (34).

### The Response of Leukemic Cells to C3 and C5 Cleavage Fragments

The role of the ComC in solid tumor malignancies has already been the subject of several extensive studies. It is also well known that the C3 cleavage fragments (C3a and C5a anaphylatoxins) directly promote migration of normal differentiated hematopoietic cells, including leucocytes, monocytes, lymphocytes, and NK cells. The additive role of ComC cleavage fragments in co-regulating migration of normal HSPCs was presented earlier in this review. However, as mentioned above, in contrast to normal human hematopoietic cells, there is relatively little evidence concerning ComC involvement in leukemia, and there are limited reports on the expression of C3aR and C5aR by leukemic cells. It has been demonstrated, for example, that the HL-60, THP-1, and U-937 cell lines express both functional receptors and that their expression is regulated by interferon gamma (IFN-γ) and phorbol myristate acetate (35–40).

To fill in this knowledge gap, we analyzed seven myeloid (HEL, K-562, THP-1, U937, KG-1a, HL-60, DAMI) and five lymphoid (DAUDI, RAJI, NALM-6, JURKAT, MOLT4) cell lines as well as primary CD33^+^ AML and CML patient leukemic blasts to see whether they express C3aR and C5aR, according to RT-PCR (17). The expression of these receptors was subsequently evaluated at the protein level by FACS. We also asked whether release of C3a and C5a anaphylatoxins due to ComC activation affects the biology of these cells and whether C3aR and C5aR are functional on the surface of leukemic blasts (17).

We found that all cell lines evaluated in our studies expressed mRNA for both receptors, except for K-562, which does not express C3aR, and DAMI and JURKAT cells, which do not express C5aR. The expression of C3aR and C5aR mRNA was corroborated by expression at the protein level by FACS. We also found expression of C3aR and C5aR receptors on the surface of CD33^+^ blasts purified from CML and AML patients. Both receptors were expressed at the mRNA and protein levels (17).

To assess the functionality of C3aR and C5R, we stimulated cells with C3a or C5a and evaluated the effect on proliferation, survival, migration, and adhesion of the tested leukemic cell lines. First, we evaluated the potential effect of these anaphylatoxins on proliferation of leukemic cells by adding C3a or C5a to liquid cultures of leukemic cells or by adding both ComC cleavage fragments to clonogenic colony-forming units of granulocyte-monocyte (CFU-GM) assays of CD33^+^ blasts isolated from patient AML or CML cells. While C3 and C5 cleavage fragments were reported to stimulate proliferation of some solid cancer cell lines, in our hands we did not observe any effect of C3a and C5a on proliferation of human leukemic cell lines. Similarly, C3a or C5a also did not affect survival of leukemic cells in serum-free cultures supplemented with 0.5% bovine serum albumin (17).

As mentioned above, C3 and C5 cleavage fragments directly stimulate migration of normal differentiated hematopoietic cells. Therefore, we next became interested in the role of C3a and C5a anaphylatoxins in regulation of the migration of human leukemic cell lines. Again we performed Transwell migration assays with established human cell lines and CD33^+^ blasts isolated from AML and CML patients. We found that C3a and C5a induced migration of human leukemic cell lines and clonogenic AML and CML blasts. Thus, our data indicates that the responsiveness of clonogenic leukemic cells to C3a and C5a is most likely a result of their malignant transformation (17).

Since the motility of cells in response to migration stimulatory factors may be the result of either gradient-orchestrated unidirectional cell movement (chemotaxis) or random migration (chemokinesis) (41), we tested whether the effect of C3 and C5 cleavage fragments (C3a and C5a) on the migration of human leukemic cell lines in a Transwell assay depends on one process or the other. We found that chemokinesis is the main phenomenon responsible for the enhanced migration of leukemic cells. Moreover, we also observed C3aR and C5aR expression-mediated adhesion of our leukemic cell lines to fibronectin-coated plates. Our receptor expression results and the observed cell responsiveness in migration and adhesion assays corresponded with activation of the p44/42 MAPK and AKT signaling pathways (17).

### The Molecular Basis of ComC-Mediated Migration of Human Leukemic Cells

Evidence has accumulated that activation of the ComC is negatively regulated by the anti-inflammatory effect of HO-1, and, *vice versa*, ComC activation leads to downregulation of HO-1 in the cells (16, 42, 43). HO-1 is an inducible anti-inflammatory enzyme that is upregulated in response to several oxidative stress stimuli, and the anti-inflammatory functions of HO-1 have been very well-demonstrated in HO-1 knockout (KO) mice as well as in rare cases of human HO-1 deficiency. These *in vivo* HO-1 deficiencies provide evidence that HO-1 somehow balances the effects of ComC activation. Moreover, it has been reported that HO-1 is also a negative regulator of cell motility (16, 42). Downregulation of HO-1 inside cells leads to enhanced migration, whereas upregulation of HO-1 has the opposite effect. This process involves p38 MAPK, which negatively regulates intracellular HO-1 expression (44, 45).

To better understand this phenomenon, we stimulated two human leukemic cells lines, U937 and KG-1a, with C3a or C5a and confirmed that these pro-migratory factors downregulate the expression of HO-1 at the mRNA and protein levels in leukemic cells by upregulating p38 MAPK. This finding suggests the possibility of inhibition of the *in vivo* spreading of leukemia cells by intracellular inhibition of p38 MAPK and/or upregulation of HO-1 expression (17, 42).

To address this issue, we downregulated expression of p38 MAPK in U937 and KG-1a cells by employing the specific small-molecule inhibitor SB203580, and, as expected, SB203580 was a potent inhibitor of leukemic cell migration in response to C3a, C5a, or SDF-1 gradients in Transwell chemotactic assays. A similar effect on the migration of leukemic cells was obtained by upregulation of HO-1 activity by exposing cells to the HO-1 small-molecule activator CoPP (17). Thus, activation of the ComC in leukemia/lymphoma patients (e.g., as the result of accompanying microbial infections or chemotherapy-induced sterile inflammation) and release of C3 and C5 cleavage fragments could be ameliorated by inhibition of p38 MAPK or upregulation of HO-1. Such a treatment strategy would have a beneficial effect on decreasing the risk of *in vivo* spread of leukemia/lymphoma cells. The efficacy of this potential therapeutic approach has been confirmed in immunodeficient mice injected with human leukemia cells. Another therapeutic possibility for inhibiting ComC activation would be application of ComC inhibitors (e.g., compstatin) or small-molecule inhibitors of the inflammasome (e.g., MCC950) (46).

### Modulation of the ComC in Leukemia Patients

Both extracellular activation of the ComC and, very likely, also its intracellular activation (a complosome effect) plays a pleiotropic role in leukemia development as a link between the tumor and the host immune system (22–24). Specifically, this mutual relationship regulates tumor growth in different ways. On the one hand, it is well-known that activation of the ComC is an important element in antibody-dependent cellular toxicity, complement-dependent cytotoxicity, and the clearance of apoptotic cells (47, 48). Elements of the ComC may protect tumor cells from NK attack by membrane-bound or soluble regulators (e.g., CD55, CD59, and factor H) and by suppression of anti-tumor T cell immunity. However, more studies are needed to assess the role of complosome activation in leukemia blasts and in cells involved in a potential immune response.

These unwanted effects of ComC activation could be ameliorated by C3aR and C5aR inhibitors as well as inhibitors of membrane-bound and soluble regulators. One may also consider the clinical application of more general ComC inhibitors, such as eculizumab or compstatin, to inhibit the C3a- and C5a-mediated spread/dissemination of leukemia cells (49). In addition, some small molecules that upregulate expression of HO-1 and inhibit p38 MAPK could also potentially find practical application, and our *in vivo* results lend support to this possibility. Of note, inhibition of p38 MAPK has already been employed in the clinic to inhibit progression of myelodysplastic syndrome (MDS) and to improve hematopoiesis in MDS patients (50).

Based on the scheme shown in Figure 2, activation of the ComC in leukemia patients could potentially be inhibited by employing inhibitors of inflammasome activation or by inhibiting ATP-mediated activation of the P2X7 receptor (51, 52). These possibilities, however, require further study, as targeting these mechanisms may lead to unwanted side effects.

## Conclusions

In conclusion, leukemia cell lines employed in our studies, both myeloid and lymphoid, as well as clonogenic blasts isolated from AML and CML patients express functional C3aR and C5aR receptors, and as we reported in the past leukemic cells respond to stimulation by these anaphylatoxins by an enhanced random migration known as chemokinesis (17). This is a relevant phenomenon to all the situations in which leukemic cells become exposed to active ComC fragments. Both sterile inflammation induced by chemotherapy or radiotherapy and accompanying microbial-induced infections in leukemia patients enhance the migratory potential of malignant blasts, and this enhanced motility may contribute to the systemic spread of leukemic cells. This process is mediated by downregulation of HO-1 in leukemic cells in a p38 MAPK-dependent manner. Therefore, inhibition of this axis by employing activators of HO-1 or inhibitors of p38 MAPK may have a beneficial effect in ameliorating this unwanted phenomenon. This has to be balanced by a better understanding of the role of the ComC in enhancing the migration of leukemia cells and, on the other hand, in its potential involvement in the immune response to leukemia. More studies are also needed to understand the role of the ComC in regulating other aspects of leukemogenesis, such as the potential involvement of intracellular autocrine C3 activation (involving the complosome). Finally, at a mechanistic level, we propose that both chemotherapy and radiotherapy activate a purinergic signaling–inflammasome–ComC axis and lead to the occurrence of sterile inflammation in collateral tissues.

## Author Contributions

MR conceived idea and wrote a paper. AL generated data for Figure 4. KB generated data for Figure 1. BX generated data for Figure 3. KB-J and other authors contributed to writing manuscript and approved it.

### Conflict of Interest Statement

The authors declare that the research was conducted in the absence of any commercial or financial relationships that could be construed as a potential conflict of interest.
