# Supplementary material for: The Complement Cascade as a Mediator of Human Malignant Hematopoietic Cell Trafficking
Source: Front Immunol. 2019 Jun 7;10:1292. doi: 10.3389/fimmu.2019.01292 (PMC6567995; doi:10.3389/fimmu.2019.01292)
Supplement: Supplementary file 1 [file Table_1.DOCX]

**Reverse transcription polymerase chain reaction (RT-PCR) for detection of C3 and C5 mRNA expression**

Total RNA was extracted and purified from various myeloid and lymphoid leukemia cell lines using the RNeasy Mini kit (Qiagen Inc., Valencia, CA, USA). The purified mRNA (500ng) was reverse-transcribed into cDNA using First Strand cDNA Synthesis Kit (Thermo Scientific, Waltham, MA, USA) according to the manufacturer's instructions. Synthesized cDNA fragments were amplified using rTaq polymerase (TaKaRa Bio Inc., Kusatsu, Shiga Prefecture, Japan) and human sequence-specific primers:

C3:

* forward primer: 5′-TCGTTTCCCGAAGTGAGTTCC-3′

* reverse primer: 5′-GATTCCATTTTTCGGTGGCTCT-3′

C5:

* forward primer: 5′-AGCGAGCTGCACGGATTAGTT-3′

* reverse primer: 5′-GCT TGCGACGACACAACATTC-3′

β-actin:

* forward primer: 5′-GGATGCAGAAGGAGATCACTG-3′

* reverse primer: 5′-CGATCCACACGGAGTACTTG-3′

The PCR conditions were: 1 cycle of 1 min at 95 °C; 40 cycles of 30 s at 95 °C, 1 min at 60 °C, and 1 min at 72 °C; and 1 cycle of 10 min at 72 °C. Samples without template controls and reverse transcriptase were used in each run. All PCR products were analyzed by 2% agarose gel electrophoresis.

**Real-time quantitative polymerase chain reaction to evaluate the expression of inflammasome genes**

Mice were injected intraperitoneally with one dose of vincristine (0.5 mg/kg). PB was obtained from the vena cava (with a 25-gauge needle and 1-ml syringe containing 250 U heparin) at 1, 12, or 24 h after the vincristine injection. Total RNA from PB was isolated using the RNeasy Mini Kit (Qiagen Inc., Valencia, CA, USA), while messenger RNA was reverse transcribed with iScript (Bio-Rad). The resulting cDNA fragments were amplified using the SYBR Green system (Applied Biosystems, Carlsbad, CA, USA). Primer sequences for the genes encoding β2 microglobulin (β2m), Nlrp3, Asc, caspase 1, interleukin 1β, interleukin 18, Hmgb-1, and S100a9 (calgranulin B) are as follows:

β2m

* forward primer: 5′-ATGCTATCCAGAAAACCCCTCAAAT-3′

* reverse primer: 5′-AACTGTGTTACGTAGCAGTTCAGTA-3′

Nlrp3

* forward primer: 5′- ACCAGCCAGAGTGGAATGAC -3′

* reverse primer: 5′- ATGGAGATGCGGGAGAGATA -3′

Asc (also known as Pycard)

* forward primer: 5′- GCCAGAACAGGACACTTTGTG -3′

* reverse primer: 5′- AGTCAGCACACTGCCATGC -3′

Casp1

* forward primer: 5′- GCTTTCTGCTCTTCAACACC -3′

* reverse primer: 5′- AAAATGTCCTCCAAGTCACAAG -3′

Il-1β

* forward primer: 5′- AGTTGACGGACCCCAAAAG -3′

* reverse primer: 5′- CTTCTCCACAGCCACAATGA -3′

Il-18

* forward primer: 5′- ACAACTTTGGCCGACTTCAC -3′

* reverse primer: 5′- GTCTGGTCTGGGGTTCACTG -3′

Hmgb1

* forward primer: 5′- GGAGGAGCACAAGAAGAAGC -3′

* reverse primer: 5′- GGGGGATGTAGGTTTTCATTT -3′

S100a9

* forward primer: 5′- TGGTGGAAGCACAGTTGG -3′

* reverse primer: 5′- CATCAGCATCATACACTCCTCAA -3′

The relative value of the target, normalized to an endogenous control gene (β2m) and relative to a calibrator, is expressed as 2^–ΔΔCt^ (fold difference), in which ΔCt equals the Ct of the target gene minus the Ct of the endogenous control gene (β2m), and ΔΔCt equals the ΔCt of the samples for the target gene minus the ΔCt of the calibrator for the target gene. To avoid the possibility of amplifying DNA contamination, uniform amplification of the products was rechecked by analyzing the melting curves of the amplified products (dissociation curves). It was found that the melting temperature (Tm) was 57–60 °C, while the product Tm was at least 10 °C higher than the primer Tm.
